# Analysis of the resistance mechanisms in sugarcane during *Sporisorium scitamineum* infection using RNA-seq and microscopy

**DOI:** 10.1371/journal.pone.0197840

**Published:** 2018-05-24

**Authors:** Meredith D. McNeil, Shamsul A. Bhuiyan, Paul J. Berkman, Barry J. Croft, Karen S. Aitken

**Affiliations:** 1 CSIRO Plant Industry, Queensland Bioscience Precinct, St Lucia, QLD, Australia; 2 Sugar Research Australia Ltd (SRA), Woodford, Australia; Stony Brook University, UNITED STATES

## Abstract

Smut caused by biotrophic fungus *Sporisorium scitamineum* is a major disease of cultivated sugarcane that can cause considerable yield losses. It has been suggested in literature that there are at least two types of resistance mechanisms in sugarcane plants: an external resistance, due to chemical or physical barriers in the sugarcane bud, and an internal resistance governed by the interaction of plant and fungus within the plant tissue. Detailed molecular studies interrogating these two different resistance mechanisms in sugarcane are scarce. Here, we use light microscopy and global expression profiling with RNA-seq to investigate these mechanisms in sugarcane cultivar CP74-2005, a cultivar that possibly possesses both internal and external defence mechanisms. A total of 861 differentially expressed genes (DEGs) were identified in a comparison between infected and non-infected buds at 48 hours post-inoculation (hpi), with 457 (53%) genes successfully annotated using BLAST2GO software. This includes genes involved in the phenylpropanoid pathway, cell wall biosynthesis, plant hormone signal transduction and disease resistance genes. Finally, the expression of 13 DEGs with putative roles in *S*. *scitamineum* resistance were confirmed by quantitative real-time reverse transcription PCR (qRT-PCR) analysis, and the results were consistent with the RNA-seq data. These results highlight that the early sugarcane response to *S*. *scitamineum* infection is complex and many of the disease response genes are attenuated in sugarcane cultivar CP74-2005, while others, like genes involved in the phenylpropanoid pathway, are induced. This may point to the role of the different disease resistance mechanisms that operate in cultivars such as CP74-2005, whereby the early response is dominated by external mechanisms and then as the infection progresses, the internal mechanisms are switched on. Identification of genes underlying resistance in sugarcane will increase our knowledge of the sugarcane-*S*. *scitamineum* interaction and facilitate the introgression of new resistance genes into commercial sugarcane cultivars.

## Introduction

Smut caused by a fungus *Sporisorium scitamineum* (Syd.) M. Peipenbr., M. Stoll & Oberw. (formerly called *Ustilago scitaminea*) [1], is a major disease of sugarcane worldwide that can cause considerable yield losses if susceptible varieties are planted [2, 3]. To date, the most effective management of smut is through the propagation of smut-resistant varieties [4]. In Australia in 2006, soon after smut was found in Queensland, a major program was undertaken to screen sugarcane clones from various stages of selection programs, parents and foreign varieties with the ultimate aim of replacing susceptible varieties grown in Australia with smut-resistant varieties [5]. Sugarcane resistance to smut was demonstrated to be a moderately heritable trait [6, 7] although the genetic determinants of this resistance are still unknown. Hence, to improve breeding for such varieties, the need exists for knowledge of resistance mechanisms.

Resistance of sugarcane plants to smut is thought to involve internal and external disease resistance mechanisms [8, 9]. As such, it is postulated to be a multifactorial process determined by either external or internal mechanisms, or combinations of both types. External resistance is believed to be due to a physical barrier resulting from a combination of bud structural characteristics [10], thickness or tightness of the bud scales, bud phenylpropanoids and glycosyl-flavonoids [11, 12, 13]. In the case of the external resistance mechanism, hyphae cannot penetrate through the bud scale to establish infection due to this physical barrier [14, 10]. Also, glycosidic substances from fresh bud scale affect the germination of teliospores and fail to infect the plant [11]. These substances were previously identified as flavonoids, which inhibited teliospore germination [15]. Internal resistance is expressed after the pathogen penetrates through the bud scale and is governed by several defence responses including increase in lignin concentration [16], production of glycoprotein, phytoalexins, polyamines [17, 18, 19] and a cascade of defence mechanisms induced in sugarcane by pathogen challenge that may include the induction of *R* genes [20, 21, 22, 23, 24, 25].

At present little is known about the actual nature of the internal and external defences induced by smut infection. Some varieties possess the external resistance mechanism only, some varieties have the internal resistance mechanism and some varieties may have a combination of the two resistance mechanisms [8, 26]. Smut resistance in sugarcane is quantitatively inherited and a cross between two resistant parents will produce both resistant and susceptible progeny [6] (P. A. Jackson, personal communication). For durable resistance to be achieved in a sugarcane breeding program a strategy which includes pyramiding genes that incorporate both the external and internal defence response is needed [27].

Previous studies have used various techniques that include Subtractive Suppression Hybridisation (SSH), mRNA differential display analysis and cDNA-amplified fragment length polymorphism for large-scale analysis of gene expression of sugarcane infected with smut [18, 20, 21, 22]. More recently, next-generation sequencing (NGS) has been used to simultaneously sequence a large number of different RNA molecules [28, 29]. Whole transcriptome sequencing using next-generation sequencing technologies (RNA-seq) is a convenient and rapid means to study the gene expression at the whole-genome level and define putative gene function [29, 30, 31]. Additionally, it gives an unbiased view of the transcriptome because it does not require prior knowledge of the gene sequences to be investigated and also provides information when small changes in gene expression and low-abundance transcripts are considered [32]. A number of studies have used NGS to assess the differential expression of genes due to infection with *S*. *scitamineum* in sugarcane [23, 25, 33, 34]. These studies have highlighted that the sugarcane-*S*. *scitamineum* interaction is a complex biological process. For breeding purposes, it is useful to determine the type of resistance sugarcane varieties possess, external or internal resistance mechanisms. To date, this has not been determined and this study is the first to attempt to distinguish between these mechanisms in sugarcane.

The objective of this study is to gain an insight into the different resistance mechanisms that operate in sugarcane cultivars in response to challenge by the pathogen *S*. *scitamineum* with the possibility of isolating specific gene candidates to be used in marker assisted selection in sugarcane breeding. In this study, we assessed the resistance mechanisms operating in 7 sugarcane varieties (Q117, Q99, Q142, Q208, ROC1, CP74-2005, QN80-3425) with varying levels of resistance to *S*. *scitamineum* using microscopy of the buds following infection, via dip-inoculation and wound-inoculation, with teliospores of *S*. *scitamineum*. To ascertain the genes involved in internal and external resistance, we compared the transcriptomes of uninfected buds and bud tissues infected with *S*. *scitamineum* via wound inoculation (48 h post-inoculation (hpi)) of sugarcane variety, CP74-2005. High-throughput Illumina sequencing was performed from three buds of resistant variety, CP74-2005, wound-inoculated with teliospores of *S*. *scitamineum* and three non-inoculated wounded buds. Quantitative real-time PCR (qRT-PCR) of selected genes was used to validate transcript abundance data obtained from transcriptome sequencing. Genes differentially expressed in response to infection with teliospores of *S*. *scitamineum* were identified, and the potential roles of these transcripts in internal and external resistance mechanisms in sugarcane were discussed.

## Materials and methods

### Ethics statement

Single haploid plus (+) and minus (-) spores germinated teliospores of *S*. *scitamineum* were collected from sugarcane at a commercial sugarcane field located in Bundaberg, QLD, as described by Trione [35]. The healthy buds from sugarcane varieties used to conduct the experiments were obtained from smut-free Sugar Research Australia Ltd (SRA) research sugarcane fields. No special permits were necessary for teliospores and sugarcane cultivars used, because this project was developed in collaboration with SRA researchers. This work does not involve endangered or protected species.

### Plant material, source of inoculum and inoculation

Seven sugarcane varieties with differing *S*. *scitamineum* susceptibility/resistance were used in this study: Q208, CP74-2005, Q99 (resistant control), QN80-3425, ROC1, Q142 and Q117 (susceptible control). Sugarcane stalks of each variety were stripped of all leaves and cut into single-bud setts. For each variety there were four biological replicates with three single-bud setts in each replicate. For the isolation of teliospores, mature sori were cut 10 to 20 cm below the top visible dewlap of the sugarcane plant and placed in a plastic bag. To collect the teliospores, the sorus was scraped and the material then sieved through a nylon mesh (1 by 1 mm pore size) to remove plant materials. Teliospore suspensions were made by adding 0.1g of teliospores of *S*.*scitamineum* to 100 ml of sterile deionized water with a drop of Tween-20 and mixed thoroughly with a magnetic stirrer. The spore concentration of the suspension was adjusted to 1.5 x 10^6^ teliospores mL^-1^ by counting of the teliospores in the suspension with a hemocytometer. To assess teliospore germination, the teliospores were incubated on 2% water agar at 31°C for 8h. The viability of spores before inoculation was then checked to ensure a germination percentage of >90% [36].

To investigate whether the seven sugarcane varieties possessed the external and/or internal disease resistance mechanisms, the single-bud setts were inoculated with *S*. *scitamineum* teliospore suspension by two methods [36] (i) Dip-inoculation: single-bud setts were dipped in 0.01% Tween-20 solution with or without 1.5 x 10^6^ teliospores mL^-1^ suspension for 10min [37], and (ii) Wound-inoculation: the hypodermic injection technique according to [10] was used to inoculate single-bud setts, to bypass the external resistance mechanism. A *S*. *scitamineum* teliospore suspension of 1.5 x 10^6^ spores per ml at 31°C was injected under the bud scale at the side of the bud avoiding the growing point. Other sugarcane plants were cut into single-bud setts and injected with 0.01% Tween-20 solution at 31°C (mock inoculated) as controls. All of the inoculated single-bud setts were then placed on trays of moistened vermiculite and then maintained in an illuminated germination chamber at 31°C and >80% relative humidity for a week [2]. Plants that had germinated were then planted in peat pots filled with potting mix (Searles Premium Potting Mix, J. C. & A. T. Searle Pty Ltd, Kilcoy, Qld, Australia) without fungicides, watered up to saturation and transferred to a polytunnel greenhouse. Plants were irrigated using an automated irrigation system once a day and fertilised as required [36]. For the RNA-seq experiment (CP74-2005 only) the inoculated single-bud setts were grown for 48 h and then the buds were harvested and ground in liquid N_2_ for RNA extraction. For the microscopy experiment (all 7 varieties) the inoculated single-bud setts were grown for 4 weeks before collecting the buds.

### Histopathological screen

The injected buds were sampled at 4 weeks according to the method described [15, 38]. Four plants per variety (three replicates per plant) were sampled as follows. The plant was removed from the pot and the shoot longitudinally sectioned to locate the meristematic tissue and growing tip. The growing tip was then sectioned at least 3 times to collect 1 mm thick slices, and placed into distilled water. When all sections were completed they were placed into bags and secured with string and a label added. The bags were then added to a large beaker containing enough lactoglycerol (1:1:1 Lactic acid:glycol:water), completely submerged, then heated to simmer for 5 min. The bags were removed and blotted to remove excess liquid then placed in a beaker containing 0.4% trypan blue solution for 5–7 min. The bags were again removed, rinsed three times to remove excess dye and the water blotted away. The bags were then simmered again in the lactoglycerol for a further 5 min to clear excess dye and debris. The sections were removed from the bags and examined under a microscope with 40X magnification to determine the level of colonisation. This was then scored using a scale of 0–5 (0-no colonisation, 1-highly restricted colonisation, 2-sparse colonisation<10%, 3-distributed colonisation 4- colonisation greater than 50%, 5-extensive profuse colonisation). The scores were averaged across the four plants.

### Statistical analyses

For the histopathological data, a linear mixed model was fitted to all datasets using PROC MIXED in SAS version 9.4 (SAS Institute, Cary, NC, USA) [36]. Degrees of freedom were adjusted using the Kenward–Roger method [39] and normality of residuals was tested using PROC UNIVARIATE of SAS. A logit transformation was applied to the percent of tissue colonization data prior to analysis [40] as $proportion=\frac{percent\;of\;colonisation+0.5}{100-percent\;of\;colonisation+0.5}$. Estimated logit values were then back-transformed before presenting in the result. For rating data, untransformed data were used for analysis.

Inoculation method (treatments), varieties and their interactions effects were treated as fixed effects. On the other hand, replication and the error term (residual) was treated as a random effect. For the appropriate significant factors, protected-mean comparisons of all possible pairwise differences of the means were tested at alpha = 0.01, using Fisher’s protected LSD test. PDMIX800 SAS Macro was used to convert mean separation output to letter groupings [41].

### RNA extraction, library construction and Illumina sequencing

Total RNA was extracted from 3 buds of each smut inoculated and mock inoculated CP74-2005 variety at 48 hpi using a QIAGEN Plant RNeasy mini kit (Qiagen, Hilden, Germany) according to the manufacturer’s instructions. The yield and purity of each RNA sample was checked by absorbance (Abs) at 260 and 280 nm. The integrity of all RNA samples was assessed by an aliquot of sample run on an Agilent Bioanalyser (performed by Australian Genome Research Facility Ltd (AGRF), Parkville, Victoria, Australia) with all samples with RIN score >8.80. Then 2μg of total RNA from each clone was sent to the AGRF for construction of the 6 cDNA libraries using the Illumina TruSeq RNA sample preparation kit v2 (Illumina, San Diego, CA). Briefly, poly-A tail mRNA was selected using oligo-dT magnetic beads, followed by fragmentation, cDNA synthesis, adaptor ligation, size selection of fragments and enriched by PCR to create the final cDNA library. Paired-end sequencing was performed using an Illumina HiSeq 2000 platform in a single lane (no technical reps) in accordance with the manufacturer’s instructions to generate 100bp paired end (PE) reads. The raw RNA reads were filtered by removing adapter sequences and low quality sequences through SolexaQA package 2.2 [42] using a Phred quality score of 30 and a minimum length of 75 nucleotides. The genome sequence of *S*. *scitamineum* [33] was then used to filter these RNA sequence files to remove the *S*. *scitamineum* RNA sequences from the sugarcane RNA sequences. The filtered RNA raw reads were aligned to the *S*. *scitamineum* genome assembly using SOAP [43] and any read-pairs where one or both reads aligned to the *S*. *scitamineum* genome were removed. Clean reads were used in *de novo* assembly and read-mapping to the transcriptome.

### *De novo* assembly of transcriptome

*De novo* paired-end assembly was performed to generate a non-redundant set of transcripts using the Trinity (downloaded 2012-09-25) software [44]. The filtered read pairs were normalised for kmer-coverage using the normalize_by_kmer_coverage.pl script from the Trinity package with a kmer size of 25 and coverage limit of 50, and then assembled using Trinity as a combined transcriptome of all sugarcane samples (3 biological replicates each of inoculated and mock-inoculated samples).

To assess the completeness of the *de novo* assembly, assembled sequences were validated by homology to 956 single-copy orthologous gene sets for plants using BUSCO v1.1 (Benchmarking Universal Single-Copy Orthologs) [45], downloaded from the BUSCO website (http://busco.ezlab.org/).

### Data quality control and mapping reads to reference sequence

The bioinformatic analysis was done using a local instance of the web-based Galaxy Project [46, 47, 48]. The Galaxy Project uses a web interface to cloud computing resources to bring command-line-driven tools to users without UNIX skills through the web and the computing cloud [48]. Prior to mapping the reads to the reference sequence, quality control of the data was done. FastQC (version 0.11.1) was used as a preliminary check of the RNA raw reads that the Phred scores were acceptable. In this study, an in-house alignment package, Biokanga (version 2.9.9; https://github.com/csiro-crop-informatics/biokanga), was used to map the RNA reads to the *de novo* transcriptome. Biokanga (ver 2.9.9) mapped the trimmed and filtered paired reads to the *de novo* transcriptome accepting best paired-read hits but removing non-unique alignments. Alignment parameters were set such that a maximum of two substitutions were allowed and no multi-aligned reads were accepted. Reads were aligned utilising the paired-end reads with insert sizes from 100 to 2kb. A counts matrix was then generated from the aligned files using the BAM-to-SAM matrix tool in Galaxy and used for differential expression analysis.

### Differential expression analysis and function enrichment

Transcript abundance and differential gene expression were calculated using the DESeq2 software package (ver 2.14) [49] contained in the Bioconductor software packages in the Galaxy platform for the *de novo* transcriptome aligned files [50]. As there is no annotation file available for the sugarcane *de novo* transcriptome, a command line script was written to generate a mock gtf annotation file for the annotation of differentially expressed genes. In contrast, gene expression levels were normalised in the DESeq2 software package using a normalization factor within the statistical model for differential analysis [51]. The count variance across the biological replicates was modelled using the negative binomial distribution approach in DESeq2, allowing for the identification of differentially expressed genes between the inoculated and mock-inoculated groups [52, 53]. The putative differentially expressed genes between the samples were then selected based on the expression profiles and the following two parameters: (1) the fold change between the inoculated and mock-inoculated samples was more than or equal to twofold (an absolute value of log_2_Ratio (NB/WB) ≥1) and (2) a false discovery rate (FDR) adjustment with a significance level of 0.05.

The assembled sequences identified from the DESeq2 differential gene expression analysis were BLAST aligned to the Nr (non-redundant) and Swiss_Prot databases using an *e* value of 1.00E^-5^ [52]. The BLAST result was then used for functional annotation with BLASt2GO software (ver 2.8.0) [53], including Gene Ontology (GO; http://www.geneonotology.org). BLAST hits with an *e* value <1.0E^-6^ and a GO annotation cutoff of >55 were used in the functional analyses. The annotated *de novo* transcriptome was used as a background reference for enrichment analysis with the differentially expressed genes (p-value adjusted 0.05) from DESeq2 analysis used as the test set. The Fisher’s Exact Test enrichment module was used with standard parameters.

### Validation of differentially expressed genes by quantitative real-time reverse transcription PCR (qRT-PCR)

To validate the transcriptional abundance results obtained from the RNA-seq, qRT-PCR was used. There were 13 genes chosen for validation and include: *glutathione-S-transferase (GST)*, *anthocyanidin 3-o-glucosyltransferase (A3G)*, *cinnamoyl reductase (CCR)*, *hydroxycinnamoyl-coenzyme a shikimate quinate hydroxycinnamoyltransferase (HCT)*, *Flavanone 3-dioxygenase (F3H)*, *cellulose synthase (CES)*, *cinnamyl alcohol dehydrogenase (CAD)*, *peroxidase*, *chitinase*, *germin*, *phenylalanine ammonia-lyase (PAL)*, *beta-1*,*3-glucanase*, *nucleotide-binding and leucine-rich repeat domain protein (NB-LRR) RGA4*. *Pathogenesis-related protein (PR10)*, a gene shown to be involved in resistance to *S*. *scitamineum* in sugarcane, was also selected for qRT-PCR and sequence of the primers was obtained from Peng et al. [54]. First-strand cDNA was prepared using 2 ug of each total RNA sample and SuperScript III reverse transcriptase (Invitrogen, Carlsbad, CA, USA) according to the manufacturer’s instructions. The samples used were RNA isolated from cultivars CP74-2005, Q117 and Q208 at 48 hpi with *S*. *scitamineum* (3 wound inoculated biological reps, 3 mock-inoculated biological reps for each sample). To confirm the infection of sugarcane buds at 48 hpi, primers designed to the rDNA internal transcribed spacer region (ITS1F and Rev2; S1 File) of *S*. *scitamineum* were used to amplify a 509-bp amplicon [55]. PCR was done on a 7500 Fast Real-time PCR System (Applied Biosystems, Foster City, CA, USA) with doubled-stranded DNA product synthesis monitored using SYBR Green. qRT-PCR was carried out using a SYBR Green Master Mix reagent (Applied Biosystems) in a 12 μL reaction volume containing 1μL of diluted cDNA, 200nM of each gene-specific primer set, and 2x SYBR Green Master Mix reagent. The amplification was achieved by the following protocol: 95 ^o^C for 30s, and 45 cycles of 95 ^o^C for 5 s, 58 ^o^C for 15s, and 72 ^o^C for 20s. Then a thermal denaturing cycle of 95 ^o^C for 15s and 60 ^o^C for 1 min was performed. The specificity of amplification was assessed using dissociation curves. Each sample was processed in triplicate. The reference gene used for normalising the expression signals was the actin depolymerisation factor (ADF) (GenBank accession no. CO373080) which has been shown to exhibit stable levels of expression in a broad range of sugarcane tissues [56]. The PCR efficiencies and Cq values were obtained using the LinReg PCR program [57] and relative changes in the gene expression ratios were calculated using the *X*^-ΔΔCt^ method [58], where *X* = PCR efficiency of the primers. The sequences of the genes selected for validation and the primers used are summarized in the S1 File.

## Results and discussion

Recently, transcriptomics was used to reveal the impact of *S*. *scitamineum* on sugarcane using a global approach to identify key resistance genes responding to infection in a sugarcane variety resistant to *S*. *scitamineum*. However, the mechanisms of resistance, internal or external, was not explored in these studies [23, 25, 34]. An important component of our study is to determine the type of resistance that exists in sugarcane varieties as external resistance can be overcome by physical damage to the bud (allowing the pathogen to enter the sugarcane plant). Furthermore, it has been found that ratooning increases the susceptibility of the sugarcane plant to smut and ratoon crops suffer a higher degree of infection than plant crops [59]. Currently, the phenotyping of sugarcane plants for smut resistance is via the dip-inoculation method. However, it cannot determine whether a variety possesses internal or external resistance mechanism. Bhuiyan et al. [36] suggested that by understanding the disease resistance mechanism of parent clones, sugarcane breeders should be able to formulate a breeding strategy to develop more durable smut-resistant varieties. A durable resistance could be achieved if a combination of different types of resistance mechanisms, inherited from parental clones, could be selected for and incorporated into the progeny of a cross during sugarcane breeding (ie ‘pyramiding genes’ strategy [27]). For this reason, we were interested in differentiating between the internal and external resistance mechanisms that exist in sugarcane when infected with *S*. *scitamineum*.

### Histopathological screen

We assessed the level of resistance to smut that existed in 7 sugarcane varieties; Q99, Q117, Q142, Q208A, ROC1, QN80-3425 and CP74-2005 using light microscopy following infection with *S*. *scitamineum* via two inoculation methods, dip-inoculation and wound-inoculation. This allowed us to determine the type of resistance each variety had to smut, external resistance (dip-inoculation) or internal resistance (wound-inoculation).

**Fig 1:**
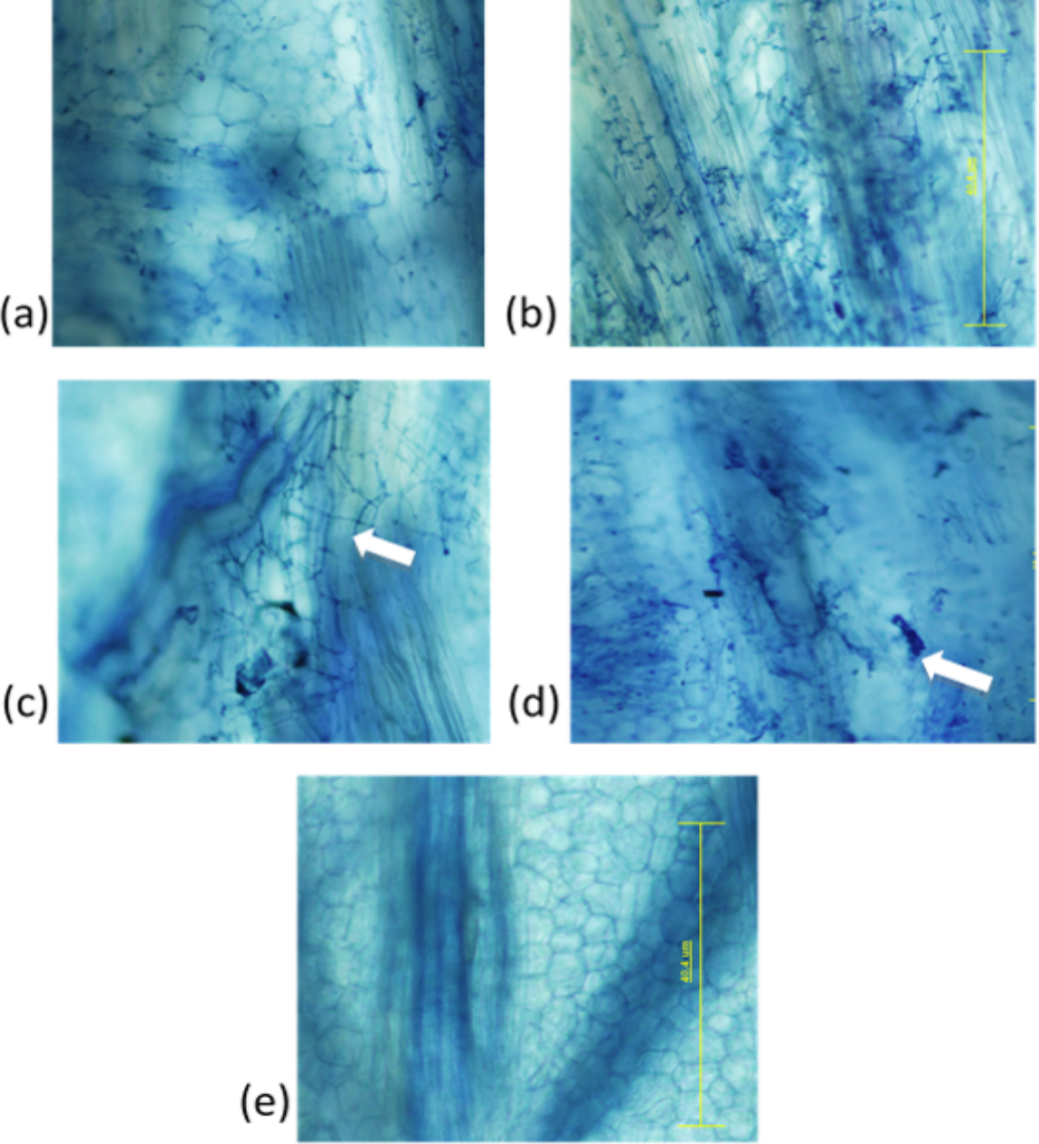
Colonization of sugarcane bud sections by *S*. *scitamineum*, **a**: bud section of Q117, 4 weeks following dip inoculation with 1.5 x 10^6^ mixed sporidia showing extensive hyphal growth, **b**: Intercellular colonisation in the meristematic cells from Q208 bud section following wound inoculation with *S*. *scitamineum*, **c**: Intracellular colonisation (arrow) in the meristematic cells from QN80-3425 bud section, **d**: Clumping of fungal hyphae (arrow) in bud section of CP74-2005 following wound inoculation with *S*. *scitamineum*, **e**: Non-detection of fungal hyphae in bud section of Q99 following 4 weeks after inoculation with 1.5 x 10^6^ mixed sporidia. Bar = 40 μm.

Microscopic examination of the meristematic tissue from buds of plants inoculated with *S*. *scitamineum* showed the presence of a network of branched fungal hyphae that was stained blue (Fig 1). In several buds such as Q117, hyphae extended from the bottom to the tip of meristematic tissue; in some other buds such as Q99 and CP74-2005, the hyphae were confined to basal portion only or around the point of inoculation. None of the meristematic tissues from the control plants showed any trace of fungal hyphae (Fig 1E). In the dip-inoculation experiments, mycelial growth was observed in sugarcane varieties Q117 (susceptible control), ROC1, Q208 and in one replicate from QN80-3425. The mycelia growth was varied with quite widespread growth in Q117 and lesser density of mycelia in ROC1 and QN80-3425. Intracellular and intercellular growth of fungal hyphae was observed (Fig 1B and 1C). The convoluted hyphae/haustoria said to be characteristic of *S*. *scitamineum* [60], was evident within cells of the meristematic tissue. *In situ*, plant cell death was not evident within the bud tissue even though the cells were heavily colonised with fungal hyphae. For the stained bud section of CP74-2005 following wound inoculation there is some minimal *S*. *scitamineum* hyphae, however, it is restricted to around the point of inoculation and there appears to be clumping of the hyphae possibly indicating the plant defences restricting the growth of the *S*. *scitamineum* in the bud tissue (Fig 1D).

Table 1 displays the rating for disease incidence for each variety in the bud section when plants were inoculated via dip-inoculation and wound-inoculation. There was a significant (P<0.0001) effect of variety and treatment on smut fungus colonisation and rating, but their interactions were not significant (data are not presented). For varieties Q117, Q208, Q142 and ROC1, there were significant (P<0.01) differences of percent of fungal colonisations or ratings between dip inoculation and wound inoculation treatments (Table 1), indicating these varieties possess external resistance mechanisms. For varieties Q99, QN80-3425 and CP74-2005, there were no significant differences of fungal colonisation or ratings observed between dip inoculation and wound inoculation treatments. That indicated they possessed internal resistance mechanisms. Varieties Q99 and CP74-2005 displayed the highest level of resistance to both inoculation methods and may possess both the internal and external resistance mechanisms. The high level of resistance to smut that CP74-2005 demonstrated in these experiments was first noted by Dean [8]. Dean carried out a number of inoculation methods using wounded and unwounded buds and determined that there were at least two components of resistance, one that is circumvented by wounding and one that is not [8]. However, it is difficult to determine if the restriction of the *S*. *sporisorium* following dip inoculation is due to restricted entry into the sugarcane bud because of the thickness or tightness of bud scales, the deposition of glycosidic substances on the bud scales (external resistance mechanism) or due to an early disease response (internal resistance mechanisms) of the plant following entry of the pathogen into the bud. It is likely that these varieties possess a combination of external and internal resistance mechanisms.

**Table 1:** Tissue colonisation (%) and disease ratings for from the histopathogical microscopy of stained cross-sections of buds from 7 sugarcane varieties, four weeks after infection with teliospores of *S*. *scitamineum* via dip-inoculation and wound-inoculation.

Variety	Tissue colonisation %		Rating^1^		Sig
	Dip inoculation	Wound inoculation	Dip inoculation	Wound inoculation	
Q117	6.8	63.7	2	3.8	**
Q208	2.9	91.9	1	4.7	**
ROC1	1.4	37.2	0.5	3.3	**
QN80-3425	1	9.7	0.5	2	ns
Q142	0.5	9.9	0	2.3	**
CP74-2005	0.5	4.1	0	1.5	ns
Q99	0.5	1.9	0	1	ns

Values are the mean of four replications. Values followed by the asterisks (**, in Sig column) in a row for tissue colonization% or ratings are significantly different according to Fisher’s protected least significant difference (LSD) test (P = 0.01), ns = not significant.

^1^Rating: 0 –no colonisation, 1 –highly restricted colonisation, 2 –sparse colonisation <10%, 3 –distributed colonisation, 4 –colonisation greater than 50%, 5 –extensive profuse colonisation. The scores were averaged across four plants.

2 Sig

** = significantly different

Our analysis demonstrates that the infection by *S*. *scitamineum* elicits visible plant defence reactions during the early stage of biotrophic development in different sugarcane varieties with differing levels of resistance to *S*. *scitamineum*. It has been reported that *S*. *scitamineum* enters the plant via the meristematic tissue of the bud 6–36 hrs after the teliospores have been deposited on the surface [61]. Also, Que et al. [23] reported that the number of genes differentially expressed following inoculation with *S*. *scitamineum* in sugarcane cultivars Yacheng05-179 (smut-resistant) and ROC22 (smut-susceptible) at 48 hpi was higher than genes differentially expressed at 24 and 120 hpi. Therefore, as we wished to detect the early response to infection with *S*. *scitamineum* in sugarcane, we collected RNA for RNA-seq analysis 48 hpi. As the time point and varieties used in this microscopic study provided a view of the fungal development as well as the plant response for internal and external resistance to *S*. *scitamineum* in sugarcane, we have chosen one variety, CP74-2005 (that possibly possesses internal and external resistance), and one time point (48 hpi) for our subsequent RNA-seq studies. As there were no visible symptoms at 48 hpi in the CP74-2005 buds, we used primers to amplify a 509-bp sequence that corresponds to the 5.8S ribosomal RNA gene (that flanks internal transcribed spacers 1 and 2 in *S*. *scitamineum* [55]) to confirm fungal infection (S2 File). This was also confirmed in buds collected a 48 hpi for cultivars Q117 and Q208 which were used in the qRT-PCR validation analysis of genes identified in the RNA-seq differential expression analysis (S2 File).

### Analyses of RNA-seq data: Read number, transcriptome coverage and total expressed genes

The absence of an annotated sugarcane genome hampers the study of the actual disease resistance mechanism in the *S*.*scitamineum*-sugarcane system. Large scale transcriptomics has evolved to be a very useful technique for providing large expression data in much shorter time period, depth and coverage to expedite understanding of metabolic pathway as well as contribute to comparative transcriptomics, evolutionary genomics, and gene discovery [62]. Recent studies, using RNA-seq to study the *S*. *scitamineum*-sugarcane interaction, have demonstrated a general disease response to *S*. *scitamineum* infection in sugarcane such as, plant hormone signal transduction, flavonoid biosynthesis, plant-pathogen interaction, cell wall fortification pathway and other resistance-associated metabolic pathways [23, 33, 34]. Que et al. [23] showed *chitinase* genes were differentially expressed in susceptible and resistant cultivar infected with *S*. *scitamineum*. They also showed that differentially expressed genes increased with different time-points following infection. Recently, Su et al. [63] linked proteomic data and RNA-seq data to identify differentially expressed proteins in susceptible and resistant cultivars infected with *S*. *scitamineum* at 48 hpi. They showed that ethylene and gibberellic acid pathways, phenylpropanoid metabolism, peroxidase, beta-1,3-glucanase and pathogenesis-related proteins had possible roles in sugarcane smut resistance. With this background, the work was initiated with an aim of identifying and characterising genes involved in the different internal and external disease resistance mechanisms of sugarcane to infection with *S*. *scitamineum*.

For a comprehensive analysis of host cell responses, we performed transcriptome profiling using RNA-seq analysis on sugarcane variety CP74-2005 following inoculation with *S*. *scitamineum*. The aim was to develop a model of internal and external disease resistance mechanisms operating in resistant sugarcane variety, CP74-2005. Buds from CP74-2005 were infected with *S*. *scitamineum* via wounding and RNA collected at 48 hpi. Changes in gene expression were calculated relative to control plants inoculated with water.

**Fig 2:**
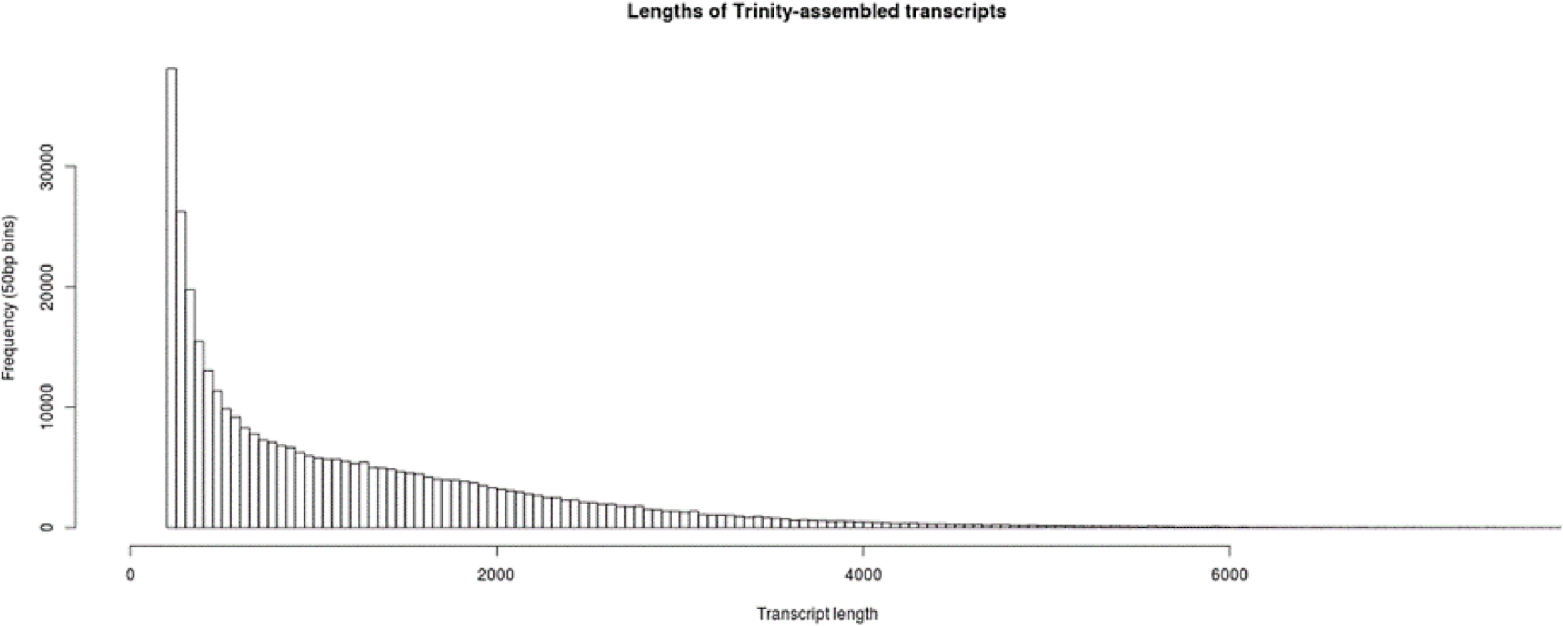
Lengths of the assembled transcripts from Trinity for the *de novo* transcriptome.

The Trinity assembly of the reads from all samples resulted in a total of 318,762 isoforms of 138,062 genes. The total size of the assembled genes in the transcriptome was 87,204,983 bp, while the total size of the assembled isoforms was 433,696,444 bp. 59.1% of sequences were over 500 bp in length while 37.4% of sequences were over 1 kbp in length with an N50 of 1,758 bp for the assembled isoforms (Fig 2). One of the biggest challenges in sugarcane is to accurately assemble short reads from this non-model organism into a *de novo* transcriptome assembly without the aid of a reference genome. The resultant large number of gene transcripts identified is likely to be a result of identifying splice variants, gene fusion events, the polyploid nature of the sugarcane genome and also the creation of chimeras that are the result of misassembled short reads or PCR-induced recombination during library preparation [64, 65].

To evaluate the accuracy of assembled transcripts, alignment of reads to the reference genome is an important step for assembly quality reliability of transcripts. However, due to the lack of genome information for sugarcane, the *de novo* transcriptome was used to ensure the correct identification of genes differentially expressed due to infection with *S*. *sporisorium* in sugarcane. During alignment, a total of 37,119,100 read pairs (42.6%) from all of the samples could be mapped to the *de novo* transcriptome. The failure to align the remainder of the reads could be explained due to the ploidy of the sugarcane genome such that there was a higher sequence divergence between reads and reference than the similarity cut-off (maximum of 3 polymorphisms per read used as an alignment parameter). For a further assessment of the quality of the assembled transcriptome we performed BUSCO analysis, a recognized benchmark approach for single-copy orthologs providing an assessment of orthologs conserved among species [45]. BUSCO provides measures for the quantitative assessment of transcriptome completeness based on evolutionary informed expectations of gene content from near-universal single-copy orthologs selected from OrthoDB [66]. We searched the transcriptome for the presence or absence of a list of conserved orthologous genes using the BUSCO library of 956 single-copy plant genes. Table 2 reports the BUSCO notation assessments for the *de novo* transcriptome assembly. We obtained a BUSCO completeness score of 91% indicating that the assembled transcriptome contained a high representation of single-copy orthologs that are in the BUSCO plant gene set. There was also a high level of duplication (61%) shown in the transcriptome which is to be expected for a polyploid genome such as sugarcane.

**Table 2:** Assessment of *de novo* transcriptome assembly in BUSCO notation.

**Size**	**BUSCO notation assessment results**
138, 062 genes	C:91% [D:64%], F:5.4%, M:2.8%, n:956

(C:complete [D:duplicated], F:fragmented, M:missing, n: gene number).

### Response to *S*. *scitamineum* inoculation

**Fig 3:**
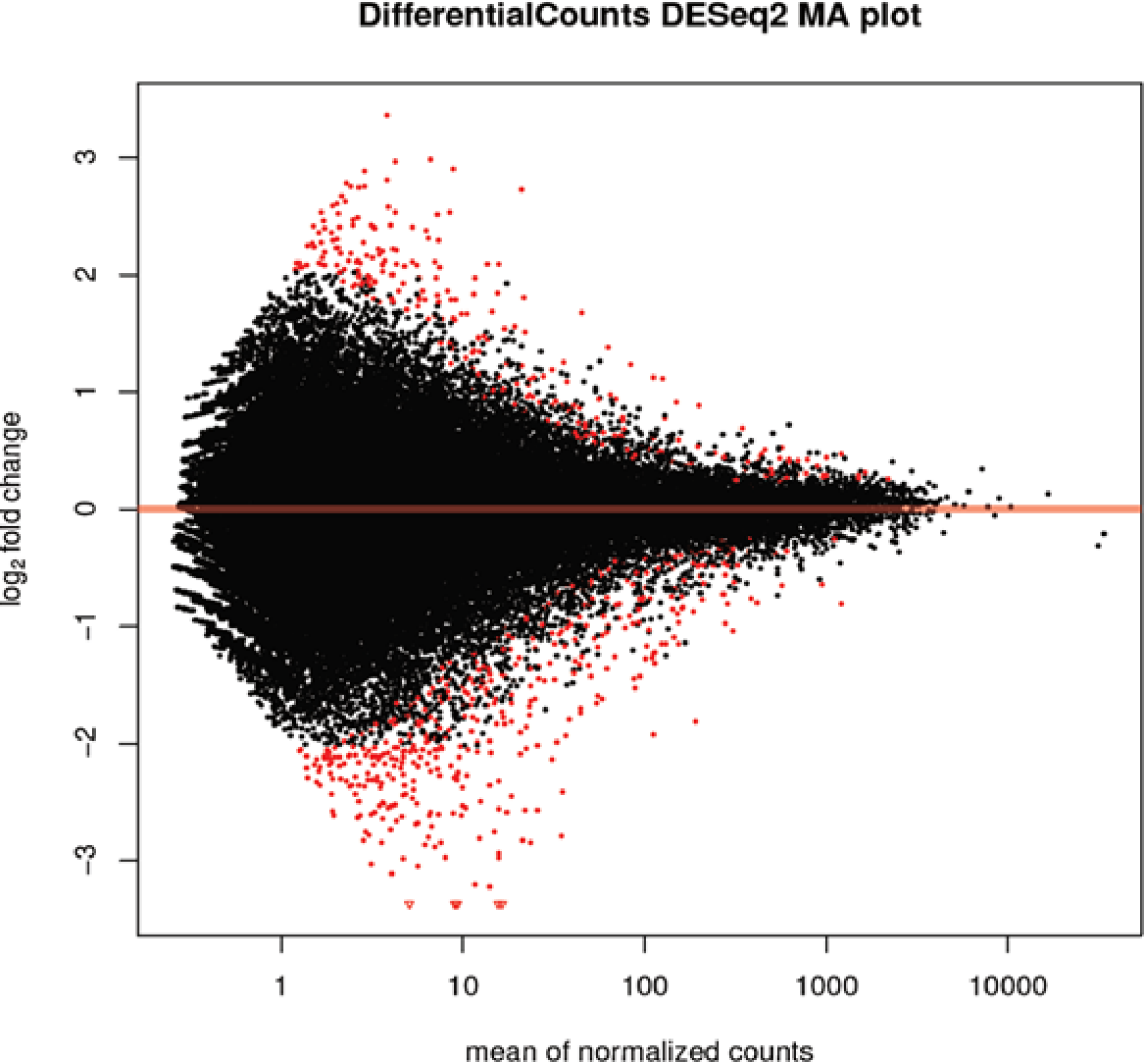
Volcano plot generated from DESeq2 software of gene expression levels of smut-inoculated and mock-inoculated samples. The differentially expressed genes are shown in red. Fold change ≥ 2, FDR < 0.05.

To identify differentially expressed genes (DEGs) in the bud tissue of CP74-2005 that were specifically induced or suppressed in response to infection by *S*. *scitamineum*, we used the DESeq2 package in the Galaxy platform to evaluate the significance of differences in expression and to control for false discovery rate (FDR). The P-values generated from the DESeq2 analysis were adjusted for false discovery rates (FDR) across the multiple tests by using the procedure of Benjamini and Hochberg as implemented in the DESeq2 package [67]. The putative differentially expressed genes were selected by performing a pair-wise comparison of the expression profiles of the inoculated and the mock-inoculated samples at 48 hpi in a two-step process. The first step was to categorise expressed genes according to the false discovery rate (FDR) value that was less than or equal to a significance level of 0.05. At this stage, 861 genes were differentially expressed due to the inoculation of the fungus; 40% (343) of them were up-regulated and 60% (518) of them were down-regulated in the *de novo* analysis (S3 File). The second step then looked at the average fold change between the smut-inoculated and mock-inoculated genes that was more than or equal to twofold. Using this criterion, a total of 497 (58%) genes were differentially expressed due to the inoculation of the fungus; 46% (231) of them were up-regulated and 53% (265) were down-regulated in the *de novo* analysis. We also used MA plot analysis to examine the magnitude distribution of the significantly regulated genes comparing the expression level to the log-transformed fold-change between inoculated and mock-inoculated samples (Fig 3).

#### GO annotation of differentially expressed genes

**Fig 4:**
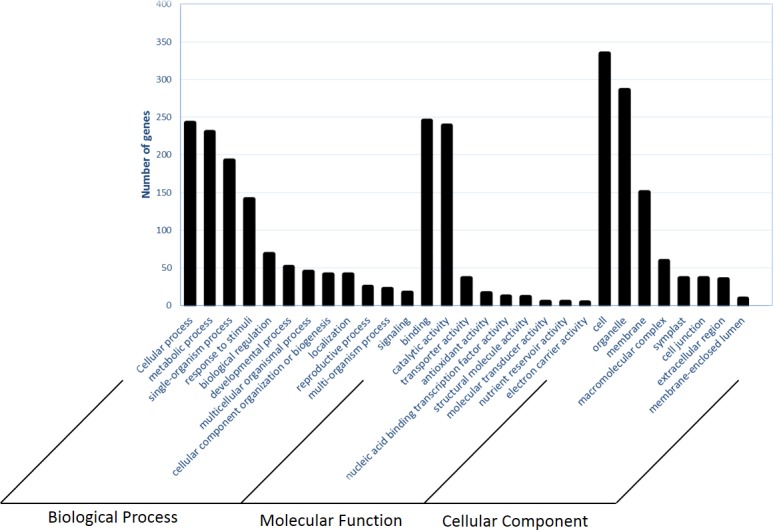
Functional classification of the 861 differentially expressed genes in CP74-2005 within the GO secondary categories of biological process, molecular function and cellular component at 48 h post inoculation (hpi) with *S*. *scitamineum*.

We used GO enrichment analysis of the 861 genes identified in the differential expression analysis to classify the biological function of these genes induced by infection with *S*. *scitamineum* in sugarcane (Fig 4). Of these 861 differentially expressed genes, 457 (53%) sequences were successfully annotated, with 322 (62%) annotated sequences for the down-regulated genes and 135 (39%) annotated sequences for the up-regulated genes using Blast2GO software. Generally, more genes were assigned to the biological process and cellular component categories than in the molecular function category. The distribution of the GO functions revealed that “metabolic process” (23.2% up-regulated genes; 19.1% down-regulated genes), “cellular process” (24.7% up-regulated genes; 20.1% down-regulated genes) and “response to stimulus” (12.6% up-regulated genes; 11.8% down-regulated genes) were the most represented secondary categories in the biological processes at 48 hpi which may indicate that the defence mechanisms of the sugarcane plants were activated by the pathogen at or before 48 hpi. In the category of cellular components, a higher proportion of GO terms were associated with cell, organelle and membrane components. In the category of molecular functions, a higher proportion of genes were involved in catalytic and binding activity. The Fisher’s exact test [68] was used to determine which gene ontology (GO) terms were enriched in our dataset of 861 differentially expressed genes. The *de novo* transcriptome was used as the reference set and annotated using BLAST2GO, annotations were assigned to genes representing roughly half of the sequences within the reference set. When the 861 genes were queried against the *de novo* transcriptome reference set, enriched GO categories were associated with binding, membrane, oxidation-reduction process, oxidoreductase activity, lipid transport, peroxidase activity (S4 File). As the point of pathogen contact, cell membranes play a primary role in recognizing pathogens and triggering a defence response [69].

As previously discussed, resistance to smut is thought to be a multifactorial process determined by combinations of bud structural characteristics, bud phenylpropanoids and glycosyl-flavonoids [10, 12, 13, 70] and a cascade of defence mechanisms induced in sugarcane by pathogen challenge that may include the induction of *R* genes [27, 71]. Pre-formed plant barriers (passive defence mechanisms/external mechanisms), such as cell walls, or in the case of sugarcane thick or tight bud scales, antimicrobial compounds and other secondary metabolites is often referred to as the first line of plant defence against pathogens [72, 73]. Following entry of the pathogen into the host cells, the second obstacle the pathogen will face is the inducible plant defence responses (active defence mechanisms/internal mechanisms) that includes the classical gene-for-gene interactions between pathogen avirulence (*avr*) genes and the plant resistance (*R*) genes [73, 74]. Plants recognize general elicitors from pathogens in a nonspecific manner to activate defence responses [74]. Some of the plant defence responses that are induced because of general elicitors and Pathogen-Associated Molecular Patterns (PAMPs) include cell wall thickening, cell wall lignification, accumulation of phenolics, production of saponins and phytoalexins, papilla formation and induction of *PR* genes [75, 76].

The microscopy study indicated that CP74-2005 possibly possessed both the external and internal resistance mechanisms and we expected that we may detect genes involved in both resistance mechanisms in the RNA-seq analysis. The infection of *S*.*scitamineum* in CP74-2005 was by injecting the pathogen directly into the sugarcane bud, thereby bypassing some of the innate external resistance mechanisms of the plant, this includes the tight bud scales that may inhibit the ability of the pathogen to enter the plant. However, this may also trigger some of the genes such as the deposition of glycosidic substances on the bud scale to chemically prevent teliospore germination [11, 15] and further entry of the pathogen into the plant.

Our analysis of the RNA-seq data identified a number of transcripts that were up-regulated and down-regulated in infected buds and appeared to involve a wide range of biological activities (S5 File). Among the differentially expressed transcripts, a number were related to the plant-pathogen interaction. These included genes encoding proteins linked to the internal and external disease resistance mechanisms previously identified in the sugarcane-*S*.*scitamineum* system such as; signal transduction (putative leucine-rich repeat (LRR) protein kinases and transcription factors), defence (pathogenesis-related (PR) proteins and several disease-resistance proteins), hormone response (genes involved in jasmonate, abscisic acid and salicylic acid response pathways), and secondary metabolites (proteins from the flavonoid and phenylpropanoid pathways). Que et al. [23] suggested that this response to the smut pathogen in sugarcane is regulated by multi-gene networks, a finding consistent with other data which suggests that after pathogens infect plants, many metabolic pathways are affected, and gene expression in the transcription network is perturbed [77]. Overall, there was a higher number of genes down-regulated (517 genes) at 48 hpi than up-regulated (344 genes) (S3 File). A significant number of the genes expressed following induction with *S*. *scitamineum* had no known function following the Blast2GO analysis (34% of genes). The high number of down-regulated genes that we observed in sugarcane at 48 hpi may reflect the release of type III effectors by the fungal cells to suppress plant defence pathways, as has been suggested in the RNA-seq analysis of early-infected peach leaves by the invasive phytopathogen *Xanthomonas arboricola* [78]. Similarly, a microarray expression analysis of maize genes induced during *U*. *maydis* infection included a transient induction of defence response genes at 12 hpi that were repressed 24 h after the infection had been initiated [79]. Vargas et al. [80], suggested that this is a common observation for biotrophic pathogens and indicates that plant cells initially recognize and respond to the presence of the invading pathogen. However, when biotrophic pathogens such as *U*. *maydis* starts colonizing epidermal cells, the primary plant responses are attenuated [80]. Similar patterns of plant defence gene induction, followed by suppression, have been observed in other biotrophic pathosystem species [81] and the hemibiotrophic *Mycosphaerella graminicola* [82].

In general, qRT-PCR data depicted up/down regulation patterns of differentially expressed genes that were consistent with RNA-seq data results, suggesting that Illumina data are relatively reliable (S6 File). Some of the results did not show a significant difference in the relative expression of the gene between the inoculated and control samples, however, they did show a trend that corresponded with the RNA-seq data. This may be due to the polyploid nature of the sugarcane genome with the amplification of multiple alleles for the differentially expressed genes in the qRT-PCR assay being a different allele to the genes upregulated in the RNA-seq expression data. Interestingly, for the genes selected for validation with qRT-PCR, it appeared on the whole that the up-regulated genes in CP74-2005 (possible smut internal/external resistance variety) were down-regulated in Q117 (smut susceptible variety) and Q208 (smut external resistance variety) and the down-regulated genes in CP74-2005 were up-regulated in Q117 and Q208, highlighting the different resistance mechanisms that operate between these sugarcane varieties.

#### Changes in secondary metabolite synthesis during *S*. *scitamineum* infection

As mentioned, secondary metabolites such as products of the flavonoid and phenylpropanoid pathway have been implicated in the external disease resistance mechanism to *S*. *scitamineum* infection. In this study, we found that there was a similar number of secondary metabolites differentially expressed at 48 hpi (down-regulated (12 genes) and upregulated (10 genes)). Of interest, genes associated with the cell-wall biosynthesis, such as beta-expansin (beta-expansin 1a; comp92937_c1_seq1 and comp92937_c2_seq2) and cellulose synthase (cellulose synthase-like protein e6; comp106301_c0_seq3) were both down-regulated at 48 hpi. The *CES-like* gene was assayed by qRT-PCR, confirming the RNA-seq data in CP74-2005. Similarly, when assayed in Q208, this *CES-like* gene was downregulated at 48 hpi, while it was up-regulated in Q117 (S6 File). It has been observed, that sometimes knocking out essential genes involved in cell-wall biosynthesis that may result in a weakening of the cell wall, can actually lead to enhanced resistance toward specific pathogens [83]. For example, mutants of *Arabidopsis thaliana* defective in cellulose synthase (CESA) subunits showed enhanced resistance to different pathogens, including the necrotrophic fungi *Plectosphaerella cucumerina* and *Botrytis cinerea*, the vasucular bacterium *Ralstonia solanacearum*, and the biotrophic bacterium *Pseudomonas syringae* [84, 85]. The disease resistance phenotype of these mutants triggered by the collapse of the xylem vessels, was in part explained by the constitutive activation of plant immune responses such as the abscisic acid signalling pathway, rather than by alterations of the passive wall barrier [83]. In contrast, while there was a lower number of secondary metabolite genes upregulated in our study, genes important in the phenylpropanoid pathway, such as phenylalanine ammonium lyase (PAL; comp88573_c1_seq1), cinnamoyl-CoA reductase (CCR; comp105162_c0_seq1), hydroxycinnamoyl CoA: shikimate hydroxycinnamoyl transferase (HCT; comp104718_c0_seq1), flavanone 3-hydroxylase (F3H; comp101801_c0_seq7), cinnamyl alcohol dehydrogenase 8D (CAD; comp108032_c0_seq2), and anthocyanidin 3-o-glucoxyltransferase (A3G; comp101801_c0_seq7, comp100951_c1_seq1, comp72321_c0_seq1), were all upregulated indicating that this pathway may play a key role in the resistance of sugarcane to smut. Activation of the phenylpropanoid pathway is an active defence response of plants that leads to the production of chemicals with antimicrobial activities and/or as precursors of lignin/suberin for the fortification of cell walls [83, 86].

The phenylpropanoid metabolic pathway is the key metabolic pathway leading to the flavonoid pathway. Under the catalysis of PAL, phenylpropanoid produces cinnamic acid; then after catalysis of cinnamate-4 hydroxylase (C4H), cinnamic acid produces 4-coumaric acid which then yields 4-coumarate CoA under the catalysis of 4-coumarate CoA ligase (4CL). Next, under the catalysis of chalcone synthase (CHS) and chalcone isomerase (CHI), 4-coumarate CoA and its derivatives enter the downstream flavonoid and lignin biosynthetic pathways [87]. The products of these pathways subsequently represent potential phytoalexins, anthocyanins and UV protectants important in plant defence [79, 83]. Furthermore, up-regulation of lignin synthesis related enzymes, CCR and PAL would result in cell wall thickening, strengthening and lignification at the infected sites where the hypersensitive response (HR) occurred [83]. For example, in flax (*Linum usitatissimum*) cell suspension cultures treated with different fungal elicitor preparations, the expression of genes encoding PAL, CCR and CAD was elevated, PAL activity was enhanced and monolignol-derived compounds (precursors of lignin) accumulated [88]. Su et al. [63], indicated that seven proteins involved in the lignin biosynthetic pathway were induced by *S*. *scitamineum*, including CCR and CAD. We also identified a CAD (comp108032_c0_seq2; up-regulated) and a CCR (comp105162_c0_seq1; up-regulated) gene that were upregulated in CP74-2005 in the RNA-seq data. This result was confirmed by qRT-PCR for CP74-2005, while CCR was down-regulated in cultivars Q117 and Q208 and CAD was up-regulated in Q117 and down-regulated in Q208 (S6 File). Also, Schaker et al. [25] detected several transcripts related to lignin biosynthesis following infection of a smut-resistant sugarcane genotype; the expression of genes encoding HCT, CCR and peroxidase were all up-regulated after whip emission. However, the authors stated that the increase in lignin after whip emission is likely a stage in the formation of the whip, which is composed of lignified plant tissue, rather than part of the protective host response [25, 89]. In contrast, an increase in the lignification of smut-resistant plants has been detected by the overexpression of genes, PAL, C4H, 4CL and CAD in RNA-seq experiments of resistant varieties in the early moments of interaction [16, 23]. It is widely recognized that lignin provides a physical barrier against initial pathogen colonization and induced lignification is one of several plant responses to wounding and pathogen attack [88, 90]. Smith et al. [91], demonstrated that the enhanced resistance following infection by *Mycosphaerella* leaf disease of *Eucalyptus nitens*, was likely due to the deposition of lignin in infected cells which prevented the diffusion of toxins and enzymes of the pathogen into the host, as well as preventing the translocation of water and nutrients from the host cells to the pathogen.

The phenylpropanoid pathway, apart from its role in the biosynthesis of lignin, is required for the synthesis of numerous other phenolic compounds, such as coumarins, stilbenes, (neo-)lignans, flavonoids, and phenylpropanoid conjugates [92]. Of these compounds, many are considered to be phytoalexins (i.e. antimicrobial compounds) that can be implicated in plant defence [93]. Miedes et al. [83] suggested that impairing steps of the phenylpropanoid pathway can lead to either an accumulation or reduced abundance of these compounds, often resulting in pleiotropic effects on plant resistance. Konig et al. [93] demonstrated that the phenolics or the precursors of lignin contributed more to defence than lignin per se in the defence response of Arabidopsis against *Verticillium longisporum*. In our study, we also identified a number of genes involved in the phenylpropanoid biosynthesis pathway that were downregulated. Specifically, while we did detect a HCT allele upregulated to a low level (log2fold change 0.37), another HCT allele was down-regulated (comp105279_c0_seq9) at a much more significant level (log2fold change -1.57) in our analysis. It has been shown that down-regulation of HCT in Arabidopsis leads to accumulation of flavonoids and inhibition of auxin transport [70] and in alfalfa to reduced lignin levels and induction of defence responses such as the elevation of salicyclic, jasmonic and abscisic acid levels [94]. These studies show that this leads to a massive upregulation of pathogenesis and abiotic stress-related genes and enhanced tolerance to fungal infection and drought. Gallego-Giraldo et al. [94], postulated that HCT down-regulated plants exhibit constitutive activation of defence responses triggered by the release of bioactive cell wall fragments (i.e. pectic/oligogalacturonide fractions) and the subsequent production of hydrogen peroxide as a result of impaired secondary cell wall integrity. This down-regulation of HCT was confirmed by qRT-PCR for CP74-2005, while it was upregulated in Q117 and Q208 (S6 File).

Also, we observed the up-regulation of a number of *anthocyanidin 3-o-glucoxyltransferase* (*A3G*) genes. A3G is involved in the final step of the biosynthesis of anthocyanidin by conjugating anthocyanidin to sugars to regulate their bioactivity, to enhance their solubility, to protect their reactivity toward cellular oxidases, and to alter their transport properties throughout the whole plant [95]. The accumulation of anthocyanidin contributes to the response of the plant towards a number of biotic (i.e. pathogen attack) and abiotic stress (i.e. high light, waterlogging, salinity and cold stress) situations [79, 96]. As a type of flavonoid that can function as pigments with UV-protecting properties, anthocyanins can also act as antimicrobial agents as part of the plant defence system against pathogen invasion [97]. However, the precise mechanism remains unclear [98]. Snyder and Nicholson also determined that the anthocyanin pigments, apigeninine and luteonidinine, are phytoalexins of sorghum [99]. This correlates with what has been found previously in the sugarcane-*S*. *scitamineum* pathosystem that glycosidic substances isolated from fresh bud scales were found to have a linear association with smut resistance [15]. These substances were identified as flavonoids and a negative relationship between glycosidic substance content in the bud scale and resistance of sugarcane varieties to smut was observed [11], indicating that the glycosidic substance in bud scale might be a chemical (external) mechanism of resistance against infection of *S*. *scitamineum*. One of the *A3G* genes (comp100951_c1_seq1) differentially expressed at 48 hpi was assayed by qRT-PCR and confirmed the RNA-seq data for CP74-2005, however, in the Q117 and Q208 qRT-PCR this gene was down-regulated (S6 File). For Q117, the down-regulation of A3G would not provide this proposed protective effect for the plant and may contribute to the susceptibility of the plant following infection with *S*. *scitamineum*. For Q208, which possesses the external resistance mechanism it was expected that this gene may be upregulated. However, due to the polyploidy of sugarcane the other *A3G* genes that were upregulated in this study may be up-regulated in Q208 or activation of this pathway may occur earlier than 48 hpi.

Another major component, important in secondary metabolite synthesis and the regulation of key enzymes phenylalanine ammonium lyase (PAL) and chalcone synthase (CHS), is glutathione (GSH) [100, 101]. Several *glutathione-S-transferase* (*GST*) genes were induced at 48hr post-inoculation in this experiment. Five *GST* genes were induced at 48 hpi, four down-regulated and one up-regulated (S5 File). This includes the induction of gst6 homologue (comp76870_c0_seq1) that is induced upon pathogen attack in Arabidopsis [102]. Increased levels of GSH have also been shown to coincide with the induction of *PR* genes in *A*. *thaliana* [103]. GSH is an important antioxidant in plants, preventing cellular damage caused by ROS and GST which have been shown to be involved in the inactivation of cytotoxic plant metabolites and in stress responses induced by pathogen attack [104, 105]. Work by Doehlemann et al. [79] found the induction of seven GSTs 12 hpi with the maize fungal biotroph *Ustilago maydis* and speculated that the induced GSTs could be involved in scavenging oxygen radicals which also result from respiratory processes of the plant cell. Similarly, Peters et al. [24] found that GST activity was increased in a resistant sugarcane genotype infected with *S*. *sporisorium* at 6 and 12 hpi and speculated that it may contribute to the inhibition of lipid peroxidation and be associated with smut resistance. The *GST-like* gene (comp76870_c0_seq1) assayed by qRT-PCR resulted in confirmation of the CP74-2005 RNA-seq data, and was down-regulated in Q117 and Q208 indicating that it was not involved in resistance in the Q208 cultivar (S6 File).

#### Plant defence responses to *S*. *scitamineum* infection

At 48 hpi a significant amount of stress-related genes (114 genes; S5 File) were expressed in the infected plant tissue of sugarcane cultivar, CP74-2005. Also, there appeared to be a greater number of defence-related genes down-regulated at 48 hpi compared to up-regulated (86 genes compared to 28 genes; S5 File) in cultivar CP74-2005. This shows that while *S*. *scitamineum* cells were recognised, the fact that most of the defense response DEs identified were down-regulated indicates that *S*. *scitamineum* may also inhibit sugarcane defence responses early on in colonization. As mentioned previously, Doehlemann et al. [79] found that the defense response of maize plants was attenuated early (within 24 hpi) during the colonisation epidermal cells with *U*. *maydis* using transcriptional profiling. Many of the stress-related genes expressed in this study are known to be induced by abiotic stresses like wounding, however, there were a large number of induced genes that encode pathogenesis-related (PR)-like proteins [106, 107] (S5 File). Genes encoding PR proteins are often triggered during the early response to pathogen attack [107]. For example, *PR1* genes have been shown to be one of the prime marker genes in SA-signaling and described to be induced by both necrotrophic and biotrophic pathogens [108]. In sugarcane, Peng et al. [54] identified a PR protein, ScPR10 that was induced in sugarcane following inoculation with *S*. *scitamineum* and suggested it may be involved in plant defence responses to *S*. *scitamineum*. Among the differentially expressed genes we found in sugarcane cultivar CP74-2005 at 48 hpi with *S*. *scitamineum*, a number of homologs to genes encoding *PR* genes were observed. This included cytochrome P450, chitinase, NBS-LRR domain containing proteins, leucine zipper domain proteins, endo-beta glucanases and purple acid phosphatase that are all known to be involved in basal plant defence against a wide variety of pathogens [72, 109, 110] (S5 File). The five most induced *PR-like* genes encoded a beta-1,3-glucanase (PR2-like; comp496981_c0_seq1, up-regulated), a lignin forming peroxidase (PR9-like; comp39158_c0_seq1, down-regulated), pathogenesis-related protein (PR1-like; comp105990_c0_seq1, down-regulated), a germin-like protein (PR16-like; comp89484_c0_seq1, down-regulated) and chitinase (PR3-like; comp85113_c0_seq1, down-regulated). Two important hydrolytic enzymes among these PR proteins, chitinases and β-1,3-glucanases, are abundant in many plant species after infection by different types of pathogens [111]. As chitin and β-1,3-glucan are major structural components of the cell walls of many pathogenic fungi, these enzymes likely play a main role in the defence reaction against fungal pathogens by the degradation of their cell walls [107]. Transgenic studies have shown that when these two enzymes are combined, a synergic effect can usually be observed [111]. For example, tomato plants expressing tobacco class I *β-1*,*3-glucanase* and *chitinase* transgenes showed that when infected by *Fusarium oxysporum* f.sp. *lycopersici*, the plants showed an increased tolerance [112]. Su et al. [63] detected at 48 h post inoculation with *S*. *scitamineum*, at both the transcript and protein levels, an upregulation of a beta-1,3-glucanase (scGluA1) protein in smut resistant sugarcane cultivar, Yacheng05-179. Thokoane and Rutherford [20] investigated differentially expressed genes after sugarcane exposure to *S*. *scitamineum* and sequence homology analysis revealed that chitinase protein family members were induced after *S*. *scitamineum* infection after 7 d. Su et al. [113] investigated the induction of 10 *chitinase* genes following *S*. *scitamineum* infection at 24, 48 and 120 hpi. They showed different expression patterns for the *chitinase* genes and that there was a rapid response to smut pathogen inoculation at the initial stage, 24 hpi, and that it reduced at 48 hpi and then increased again at 120 hpi. Similarly, we found that enrichment analysis of DEs at 48 hpi revealed DEs encoding chitinases (3 down-regulated) and a number of β-1,3-glucanases (6 down-regulated, one up-regulated) were differentially expressed. As well, a transcriptional profiling of maize genes during *U*. *maydis* infection included a transient induction of chitinases and glucanases at 12 hpi that were repressed 24 h after the infection had begun [79].

Other *PR* genes induced at 48 hpi included two germin-like proteins (comp86851_co_seq1 and comp102962_c0_seq2), which in *Arabidopsis thaliana* are SA-induced [70], and *peroxidase-like* genes (13 *peroxidase-like* genes; S5 File). Germin-like proteins have been demonstrated to be involved in broad spectrum disease resistance to rice blast and sheath blight in rice through the enhancement of basal defense responses, specifically through H_2_O_2_ generation [114]. H_2_O_2_ is an important component of plant defence responses, such as the oxidative cross-linking of cell wall proteins and lignin precursors [115]. Reactive oxygen species (ROS) molecules, such as peroxide, are involved in the signalling for PCD during pathogen defense responses [116], and genes encoding peroxidases act in the metabolism of ROS [117]. Song et al. [118] found a number of peroxidases were expressed following infection with *S*. *scitamineum* and they speculated that the peroxidases could scavenge the excessive ROS and protect the sugarcane plants from smut pathogen infection. In contrast, we found 13 *peroxidase-like* genes were down-regulated at 48 hpi with *S*. *scitamineum*. It has been found that most necrotrophic fungal pathogens enhance ROS production to activate PCD, whereas biotrophic pathogens, such as *S*. *scitamineum*, need to minimize PCD [117, 119]. Again highlighting that in CP74-2005, the early response to *S*. *scitamineum* infection is attenuated similar to the maize-*U*. *maydis* pathosystem [79].

We selected a number of these *PR* genes for validation using qRT-PCR and included: chitinase 2-like (PR3; comp85113_c0_seq1; down-regulated), glucan beta-glucosidase (PR2, comp95195_c0_seq3; down-regulated), germin-like protein subfamily 3 (comp89484_c0_seq1; down-regulated), and peroxidase (comp39158_c0_seq1; down-regulated). We also included ScPR10 in our qRT-PCR analysis, as it is a PR protein shown to have a role in plant defense responses to infection with *S*. *scitamineum* in sugarcane [54]. The qRT-PCR results (S6 File) confirmed the RNA-seq data for these *PR* genes in CP74-2005 leading to a suppression of these genes at 48 hpi with *S*. *scitamineum*. Interestingly, when tested in Q117 (susceptible cultivar) and Q208 (external resistance) by qRT-PCR, the *chitinase* gene was upregulated in Q117 and down-regulated in Q208, genes *glucanase* and *peroxidase* were down-regulated in Q117 and up-regulated in Q208, and finally, germin was down-regulated in all three cultivars. For ScPR10, the qRT-PCR results showed that this gene was down-regulated in CP74-2005 but up-regulated in Q117 and Q208. These results suggest a general suppression of the defense response to *S*. *scitamineum* at 48 hpi in cultivar CP74-2005, with a more complex response in the other cultivars.

In many pathosystems, proteins with similar features (NBS-LRR proteins) have been described as one group of *R* genes involved in the pathogen-recognition mechanisms and defence activation [120]. Proteins that display a serine-threonine kinase (S/T KINASE) domain are another group of *R* genes that are induced during pathogen attack. Rossi et al. [121] described the identification of several resistance gene analogs (RGAs) that contain the NBS-LRR, LRR alone and S/T KINASE domains in sugarcane EST collections. Also, Que et al. [122] identified NBS resistance type proteins upregulated during the interaction between sugarcane and *S*. *scitamineum*. As a result of *S*. *scitamineum* infection, we identified a number of DE transcripts translated into proteins with NBS-LRR, LRR and S/T KINASE domains (S5 File). In particular, two DE genes up-regulated at 48 hpi, comp600089_c0_seq22 and comp95924_c2_seq2, were identified and shown to be similar to the rice *NB-LRR RGA4* gene that mediates resistance to the fungal pathogen, *Magnaporthe oryzae* [123]. Validation of this gene (comp600089_c0_seq22) by qRT-PCR confirmed that it was upregulated in CP74-2005 and Q208, but down-regulated in Q117 suggesting a role in the plant defense response to infection with *S*. *scitamineum* in these two sugarcane cultivars (S6 File).

#### *S*. *scitamineum* induced changes in hormone signalling

Plants respond to invasion by pathogens by the induction of a large number of hormones, which include jasmonates (JA), auxins, ethylene (ET), gibberellins (GA), abscisic acid (ABA), salicylic acid (SA), cytokinins (CK), brassinosteroids (BR) and peptide hormones [124]. Importantly, three of these phtyohormones (SA, JA and ET) are known to have a major role in the regulation of plant defence responses against various pathogens, pests and abiotic stresses such as wounding [124, 125]. In particular, SA chiefly mediates systemic acquired plant resistance (SAR) and the induction of pathogenesis related (*PR*) genes, which is generally associated with responses against biotrophic and hemi-biotrophic pathogens, whereas JA and ET mediate induced systemic resistance (ISR) that is usually associated with responses against necrotrophic pathogens and herbivores [126, 127].

DEs expressed in the *S*. *scitamineum*-sugarcane interaction related to hormone biosynthesis and signalling revealed that at 48 hpi, signalling mediated by the above hormones appears to be suppressed. Enrichment analysis confirmed that the auxin, JA, brassinosteroid, ethylene, abscisic acid biosynthetic process is over-represented among the down-regulated genes, with the brassinosteroid and abscisic acid group having the largest number of genes down-regulated (S5 File). ABA has been reported to affect plant responses to biotic stress mainly via interaction with other stress response pathways [128]. It is considered a negative regulatory factor in plant disease resistance, and its expression is associated with increased disease sensitivity [124]. After 48 hpi with *S*. *scitamineum* we detected 14 genes associated with the abscisic acid biosynthetic process were down-regulated with one gene, comp95124_c0_seq 2 (protein phosphatase 2C) being up-regulated (less than 2 fold). Thus, after CP74-2005 is infected by the pathogen, the ABA signalling pathway is suppressed, assisting with the resistance of the plant to the pathogen. This is similar to previous studies showing a down-regulation of PP2C transcripts following infection with *S*. *scitamineum* [23, 63]. In contrast, JA signalling acts the most rapidly to pathogen infection and is usually associated with an induction of plant defence genes like defensins, hevein-like proteins and chitinases [129]. Que et al. [23] showed that after *S*. *scitamineum* infection of a resistant sugarcane variety, a jasmonate ZIM-Domain (JAZ) and MYC transcription factor were up-regulated suggesting that *S*. *scitamineum* can stimulate the JA biosynthesis and that JA signalling pathway is involved in the response to *S*. *scitamineum*. Our study showed that while one gene implicated in the JA signalling pathway, comp96572_c0_seq2 (lipoxygenase) was up-regulated, there were four genes down-regulated at 48 hpi. Lipoxygenase (LOX) is postulated to be involved in such active resistance mechanisms as the hypersensitive response (HR), a type of programmed cell death (PCD) [130], and has been identified consistently during pathogen-induced defense responses [119, 131].

## Conclusion

In conclusion, this study provides insight into the different resistance mechanisms that operate in sugarcane when infected with *S*. *scitamineum*. This study has led to the identification of a large number of novel and known DEGs from sugarcane, and their specific modulation during resistance responses in *in vivo* infected sugarcane buds. This repertoire of genes will greatly facilitate basic and applied research on sugarcane-*S*. *scitamineum* interactions. The results presented in this study highlight that the early (48 hpi) sugarcane response to *S*. *scitamineum* infection is complex and many of the disease response genes are attenuated in sugarcane cultivar CP74-2005, while others, like genes involved in the phenylpropanoid pathway, are induced. This may point to the role of the different disease resistance mechanisms that operate in cultivars such as CP74-2005 that potentially possess both the internal and external resistance mechanisms, whereby the early response is dominated by external mechanisms and then as the infection progresses, the internal mechanisms are switched on. While with cultivar Q208, a sugarcane cultivar that the microscopy analysis showed possesses the external disease resistance mechanism, the results of the qRT-PCR analysis of a selected number of differentially expressed genes indicates a more complex defence response. This effect clearly needs to be explored further with more candidate resistance genes assayed at different time points and also with genotypes of varying levels of resistance to smut. As with all complex systems, the expression of genes in response to *S*. *scitamineum* infection is a balance between up-regulation and down-regulation genes within these networks. The ultimate aim is to uncover the different mechanisms of resistance that operate in sugarcane to enable the pyramiding of resistance genes during sugarcane breeding to ensure that a durable resistant genotype is generated.

## Supporting information

S1 File Primers used for qRT-PCR validation of selected differentially expressed genes identified from RNA-seq analysis. (XLSX)

S2 File Amplicons of primers ITS1F and Rev2 for CP74-2005, Q208 and Q117 bud samples following infection with *S*. *scitamineum* at 48 h. 1–3: CP74-2005 inoculated buds; 4–6: CP74-2005 mock-inoculated buds; 7–9: Q117 inoculated buds; 10–12: Q117 mock-inoculated buds; 13–15: Q208 inoculated buds; 16–18: Q208 mock-inoculated buds; C+ S. sporisorium DNA; C- water blank; M: 100bp DNA ladder.
(TIF)

S3 File Differentially expressed genes identified from CP74-2005 sugarcane cultivar following inoculation with teliospores of *S*. *scitamineum* or water (mock) treatment at 48 hpi, determined by RNA-seq analysis. (XLSX)

S4 File Gene ontology terms enriched within the set of differentially expressed genes as determined by the fisher exact test. (XLSX)

S5 File Gene ontology classification of up/down regulated genes in CP74-2005 sugarcane cultivar after inoculation with teliospores of *S*. *scitamineum* inoculation at 48 hpi. **A)** Differentially expressed hormone-related genes. **B)** Differentially expressed pathogenesis-related (PR) genes. **C)** Differentially expressed secondary metabolism genes. **D)** Differentially expressed primary metabolism genes. **E)** Differentially expressed plant development genes. **F)** Differentially expressed transporter genes. **G)** Differentially expressed transposable element genes. **H)** Differentially expressed unknown protein genes.
(XLSX)

S6 File qRT-PCR validation of 14 genes showing differential expression between inoculated and control samples of externally/internally resistant variety CP74-2005 (orange bars), susceptible variety Q117 (blue bars) and externally resistant variety Q208 (green bars). Y-axis: relative expression of genes, X-axis: sugarcane varieties. Symbols are ‘M’ for mock and ‘I’ for *S-*.*scitamineum* infection. The data of qRT-PCR was normalised to the *ADF* expression level. The columns represent the average relative expression ratios calculated from all three biological replications (+/- SE). **a)** *Anthocyanidin 3-o-glucosyltransferase (A3G*), **b)** *Glutathione s-transferase (GST*), **c)** *Cinnamoyl-reductase (CCR*), **d)** *Hydroxycinnamoyl-coenzyme a shikimate quinate hydroxycinnamoyltransferase (HCT*), **e)** *Flavanone 3-dioxygenase (F3H*), **f)** *Cellulose synthase (CES*), **g)** *chitinase*, **h)** *germin*, **i)** *beta-1*,*3-glucanase*, **j)** *nucleotide-binding and leucine-rich repeat domain protein (NB-LRR) RGA4*, **k)** *phenylalanine ammonia-lyase (PAL)*, **l)** *peroxidase*, **m)** *cinnamyl alcohol dehydrogenase (CAD)*, and **n)** *Pathogenesis-related protein (PR10)*, a gene shown to be involved in resistance to *S*. *scitamineum* in sugarcane, sequence of the primers was obtained from Peng et al [54].
(TIF)
