# Supplementary figures and images for: Analysis of the resistance mechanisms in sugarcane during Sporisorium scitamineum infection using RNA-seq and microscopy
Source: PLoS One. 2018 May 24;13(5):e0197840. doi: 10.1371/journal.pone.0197840 (PMC5993111; doi:10.1371/journal.pone.0197840)

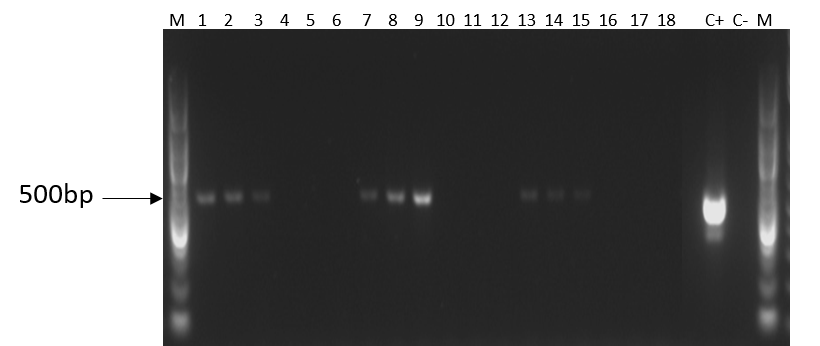

Supplement: S2 File — 1–3: CP74-2005 inoculated buds; 4–6: CP74-2005 mock-inoculated buds; 7–9: Q117 inoculated buds; 10–12: Q117 mock-inoculated buds; 13–15: Q208 inoculated buds; 16–18: Q208 mock-inoculated buds; C+ S. sporisorium DNA; C- water blank; M: 100bp DNA ladder. (TIF) [file pone.0197840.s002.tif]

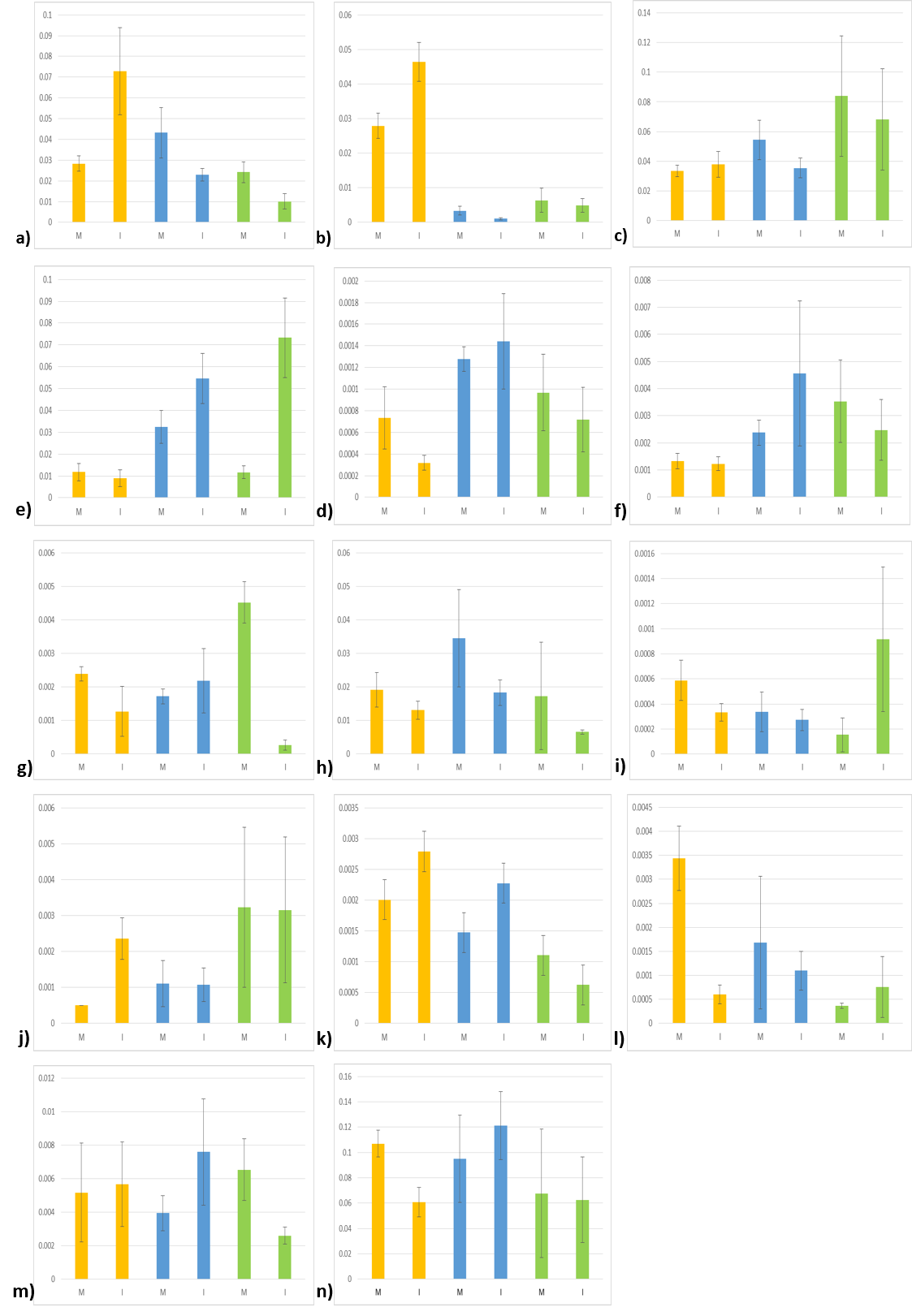

Supplement: S6 File — Y-axis: relative expression of genes, X-axis: sugarcane varieties. Symbols are ‘M’ for mock and ‘I’ for S-.scitamineum infection. The data of qRT-PCR was normalised to the ADF expression level. The columns represent the average relative expression ratios calculated from all three biological replications (+/- SE). a) Anthocyanidin 3-o-glucosyltransferase (A3G), b) Glutathione s-transferase (GST), c) Cinnamoyl-reductase (CCR), d) Hydroxycinnamoyl-coenzyme a shikimate quinate hydroxycinnamoyltransferase (HCT), e) Flavanone 3-dioxygenase (F3H), f) Cellulose synthase (CES), g) chitinase, h) germin, i) beta-1,3-glucanase, j) nucleotide-binding and leucine-rich repeat domain protein (NB-LRR) RGA4, k) phenylalanine ammonia-lyase (PAL), l) peroxidase, m) cinnamyl alcohol dehydrogenase (CAD), and n) Pathogenesis-related protein (PR10), a gene shown to be involved in resistance to S. scitamineum in sugarcane, sequence of the primers was obtained from Peng et al [54]. (TIF) [file pone.0197840.s006.tif]
